# Efficiency of Twin-Screw Extrusion of Biodegradable Poly (Butylene Succinate)-Wheat Bran Blend

**DOI:** 10.3390/ma14020424

**Published:** 2021-01-16

**Authors:** Emil Sasimowski, Łukasz Majewski, Marta Grochowicz

**Affiliations:** 1 Department of Technology and Polymer Processing, Faculty of Mechanical Engineering, Lublin University of Technology, 20-618 Lublin, Poland; e.sasimowski@pollub.pl; 2 Department of Polymer Chemistry, Institute of Chemical Sciences, Faculty of Chemistry, Maria Curie-Sklodowska University, 20-614 Lublin, Poland; mgrochowicz@umcs.pl

**Keywords:** biocomposite, twin-screw extrusion, processability, biofiller, thermal properties, chemical structure, processing characteristics, agro-waste materials, agro-flour filler, moisture content

## Abstract

Unmodified poly (butylene succinate) (PBS) is characterized by very good processability; however, after the incorporation of various fillers of plant origin, its processing becomes much more complicated and its properties are significantly affected. Detailed studies of the processing aspects of PBS/wheat bran (WB) biocomposition are lacking, despite the addition of WB having a significant impact on both the production efficiency and the properties of end products. This research paper presents test results of the co-rotating twin-screw extrusion processing of a biodegradable polymer blend, the matrix of which was PBS, with WB as the filler. In undertaking this task, we examined the impact of extruder screw rotational speed and WB content on the characteristics of extrusion processing, as well as on certain thermal, physical, structural and processing properties of the obtained blend. The WB introduced to the blend was in the form of a selected fraction with particles smaller than 0.2 mm. The measurements were conducted using the Design of Experiment (DOE) methods, which enabled establishing the studied relationships in the form of polynomials and response surfaces. The determined extrusion process characteristics covered the impact of screw rotational speed and WB content on the mass flow rate of the processed blend and its pressure, the screw drive torque and specific energy consumption. The studies of the obtained polymer blend included determining the impact of the aforementioned variable factors on the melt flow rate (MFR) index, chemical structure (FTIR), thermal properties (differential scanning calorimetry (DSC), thermogravimetry (TG), derivative thermogravimetry (DTG)), *p-v-T* relationships, microstructure, density and moisture absorbance. Analysis of variance (ANOVA) was used to assess the effect of individual variable factors. The results of this work are presented, inter alia, using Pareto charts of standardized effects, which illustrate the influence of individual terms of the determined regression equations on the studied quantity.

## 1. Introduction

The issue of the ever-increasing consumption of petrochemical polymer materials has led to the development of a significant threat to the environment. Products intended for short-term usage constitute a particular problem. For this reason, interest in utilizing biodegradable materials has increased over the last decade or so. This has resulted in the publishing of numerous papers addressing the issue of producing polymer biocomposites based on biodegradable plastics containing fillers of natural origin. Fully biodegradable and compostable polymer blends with fillers of natural origin are deemed a promising alternative to those based on traditional petrochemical rootstocks [1,2,3,4,5,6]. Biodegradable polymers commonly used for the manufacturing of biocomposites are primarily aliphatic polyesters. These are degradable by bacteria and fungi, both in soil, as well as in aqueous environments [7,8,9,10].

One of the most interesting aliphatic polyesters is poly (butylene succinate) (PBS), a white, highly-crystalline aliphatic polyester with the formula [–O–(CH_2_)_4_–O–CO–(CH_2_)_2_–CO–]_n_. PBS was commercialized relatively recently, in 1994, under the trade name of Bionolle, by Showa Highpolymer Singapore. It is the outcome of the polycondensation of 1,4-butanediol and succinic acid [11,12,13,14], and it was originally obtained solely from petrochemical substrates. One of the methods applied for obtaining 1,4-butanediol is to oxidate butane to maleic anhydride, the outcome of which is then hydrolyzed to maleic acid. This is further hydrogenated to 1,4-butanediol. In turn, succinic acid is obtained by hydrogenating maleic acid to succinic anhydride, which is further hydrated to succinic acid [15,16].

It is also possible to produce PBS from substrates coming from renewable sources. In doing this, succinic acid is obtained with the use of yeasts and bacteria, through the fermentation of sugar raw materials, such as starch, glucose or xylose [17,18,19,20]. Moreover, there are ecological methods for obtaining 1,4-butanediol. One of them involves the fermentation of maize glucose to succinic acid, which after purification through electrodialysis, is then catalytically reduced to 1,4-butanediol [16]. Despite the fact that contemporary knowledge allows for producing PBS that is derived fully from renewable sources, 46% of the content of the bioPBS currently available on the market consists of petrochemical-derived ingredients. This is because 1,4-butanediol of natural origin is commercially unavailable [21,22,23].

PBS exhibits numerous attractive properties that qualify this material for various technical applications, and which emphasize its environmentally friendly character. They include compostability and biodegradability in virtually any environment, very good processability, thermal and chemical resistance, high flexibility, high impact and tensile strength [24,25,26,27,28,29,30]. The scope of PBS mechanical properties is similar to the most commonly applied petrochemical polymers. The yield point of PBS is comparable to that of polypropylene (PP) and is approximately 2.5 times greater than that of low-density polyethylene (LDPE). The value, however, depends on the PBS grade and is around 20–45 MPa. Stiffness is between LDPE (200–300 MPa) and high-density polyethylene (HDPE) (1000 MPa). The tensile strength of PBS is higher than that of HDPE and lower than that of PP, most often in the range of 20–45 MPa. This is similar to the yield point.

In light of the above, in the early 2000s PBS was already considered to be a promising alternative to petrochemical-derived polymers, with a potential to replace them in numerous applications [13,14,16,31]. Notwithstanding the above, PBS has not been widely used to this day, and its current applications are rather niche. Certain material limitations, such as a relatively low hardness or poor gas barrier properties, contribute to this, but the direct cause is still its very high price. The cost of PBS exceeds the prices of PP and polyethylene (PE), and even that of other biodegradable materials. The reason for this is the aforementioned complex obtaining process and high substrate price. This is why, despite its favorable properties and biodegradability, PBS is used primarily in industries with high production value, e.g., the automotive industry [23,32,33,34,35].

PBS, whether of biological or petrochemical origin, can become competitive in terms of price through filling with lignocellulosic fillers (LCF) of natural origin. These are obtained at low cost or at no cost at all, and are based on process waste from the food or agricultural industries [22,36,37]. Lignocellulosic fillers can be used both in the form of fibers and powder. Depending on their origin, they differ in composition and, consequently, in properties, but their primary ingredients are cellulose, lignin, hemicellulose and starch [38,39,40]. The main restrictions in terms of using LCF are their hydrophilic nature, low strength of interfacial interactions with the matrix and their poor thermal resistance, since hemicellulose undergoes thermal decomposition around 150 °C [41,42,43,44]. PBS is a high strength material with an aliphatic structure, a low melting point of 115 °C and its particles have a hydrophilic character. This is confirmed by the contact angle at a level of approx. 70°. Therefore, it is suitable for filling with LCF up to 50 wt.% [45,46,47,48].

The source literature contains numerous studies of PBS-based biocomposites with various fillers of plant origin, such as jute [49], silk [50], sisal [51], kenaf [52] or bamboo [53] fibers, as well as ground rice hulls [31], wheat bran (WB) [54], wood [55], water bamboo hulls [13], apple [4] and grape pomace [23]. Using biofillers entails numerous advantages over mineral fillers. They exhibit lower density, are readily available and decrease friction. In addition, they do not cause rapid wear of the working elements of processing machinery [22,23,56]. Furthermore, they increase biocomposite moduli of elasticity, both in the longitudinal and transverse directions. Of note, increased strength is observed mostly in the case of fibrous fillers. Moreover, the presence of powder filler most often leads to the deterioration of mechanical properties [4,5,12,31]. Biocomposite properties largely depend on the strength of matrix/filler interface interactions and the stress transfer. Thus, many authors decide to use compatibilizers despite PBS hydrophilicity. However, the positive effect of their incorporation is relatively low, if there is one at all. Therefore, using compatibilizers with powder fillers is not economically justified [57,58,59].

Unmodified PBS is characterized by very good processability. Hence, it is suitable for extrusion and injection molding, and its grade of high molecular weight can even be used for blown film extrusion [16]. Unfortunately, the presence of LCF results in significantly lowered PBS processability due to a major increase in the melt viscosity and a decrease in the thermal resistance, but the greatest issue in the course of processing is the increased level of moisture content in the blend. Despite the hydrophilic character of PBS, the presence of water may lead to macromolecule degradation through ester bond hydrolysis, especially under the typical processing conditions of high pressure and temperature. The recommended moisture content for PBS processing should not exceed 0.1% wt., but with a high degree of LCF filling, such a moisture level is difficult or even impossible to achieve, which is a significant obstacle in designing the processing procedure [28,57,60,61].

Despite the numerous studies conducted on biodegradable materials, the available source literature does not include elaborations addressing the issue of the production and properties of PBS-based biocomposites with fillers in the form of raw wheat bran (WB), especially in terms of the processing aspects. This paper, therefore, presents a thorough analysis of the twin-screw extrusion of PBS composites with WB content in the range of 10–50wt.%. The objective of this research was to evaluate the impact of WB content and extruder screw rotational speed on the processability, thermal stability, and the physical and structural properties of a biocomposite, and to determine the characteristics of the processing itself.

## 2. Experiments

### 2.1. Test Stand

**Figure 1:**
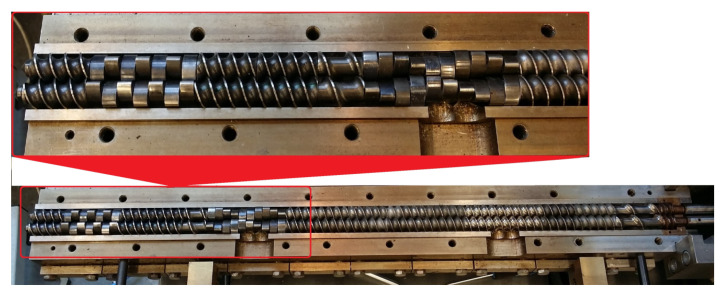
View of the extruder open plasticizing system with used processing screws.

The extrusion process was conducted using an EHP-2 × 20 Sline twin-screw co-rotating extruder manufactured by Zamak Mercator (Skawina, Poland). It was equipped with a plasticizing system with 9 heating zones, the insides of which contained segmental processing screws with a diameter of D = 20 mm, and a working section to a diameter ratio of L/D = 40. The used screw configuration is shown in Figure 1. It contained two kneading zones, the first with the double-lobe cam offset angle equal to 30°, and the second with the same equal to 90°. An extrusion head with a nozzle in the form of round openings with a diameter of 3 mm was used for the extrusion process. The obtained extrudate, in the form of two strands, was cooled in a water bath.

The process of pelletizing the obtained biocomposite strands was conducted using a G16/32 II pelletizer, again manufactured by Zamak Mercator (Skawina, Poland), which was equipped with an 18-blade rotary cutter with a diameter of 125 mm. Pelletization was executed at a composite strand collection speed of 20 m/min, and a mill cutter rotational speed equal to 320 min^−1^.

### 2.2. Materials

The prepared test samples were based on employing as matrix a PBS with the trade name BioPBS FZ91 PB [62], manufactured in the form of pellets (PTT MCC BIOCHEM CO., LTD, Bangkok, Thailand). It was synthesized using bio-based succinic acid and 1,4-butanediol. This material is intended for manufacturing general-purpose products via injection molding.

Wheat bran (WB), i.e., wheat grain shells, is a process waste product from the refining of white flour. It comes in the form of thin flakes with dimensions up to a few millimeters, and was obtained from a local mill near the city of Lublin (Poland). WB is primarily composed of fibrous substances such as cellulose, lignin and hemicellulose, but includes phytic acid, oligosaccharides, non-starch polysaccharides, as well as fats and proteins [63].

### 2.3. Research Programme and Methodology

The WB conditioning process involved, successively, two-time grinding using a millstone, drying in a laboratory drier for 24 h at 80 °C, followed by sieving the dried WB with a moisture content of 3wt.% on a 5-sieve column shaker with the mesh size decreasing from 1 mm to 0.2 mm in order to obtain a fraction with a particle size below 0.2 mm. The particle size was selected as the smallest possible on the basis of previous studies, where an adverse effect of the particle size on the properties of the composition was found [42,64].

Directly prior to use, the separated WB fraction was once again dried in a laboratory drier for 48 h at 80 °C, until moisture at a level of 1.79 wt.% was obtained (as measured via the moisture analyzer described below). Next, the dried WB, in appropriate shares pursuant to the ones included in the research plan, was mechanically mixed with PBS pellets in a planetary mixer. The resulting loose mixture, without adding any pro-adhesive agent, was directly fed to the twin-screw extruder hopper. Low processing temperatures were applied due to the risk of thermal decomposition of bran ingredients. The temperatures in all of the extruder plasticizing system heating zones were set to 145 °C, while the temperature of the extrusion head was 140 °C. The temperatures were selected based on preliminary research and source literature on PBS-based biocomposite extrusion [31,65,66]. The obtained extrudates, in the form of thin strands, were subjected to pelletization to a length of approx. 3.5 mm after drying in a laboratory dryer.

The experimental tests were conducted pursuant to the adopted central composite rotatable design, with the star-point distance of α = 1.414. The tests adopted the following independent variables—preset process conditions: extruder screw rotational speed n = 50–200 min^−1^, WB content introduced into the PBS u = 10–50 wt.%. The composition of the blend and its processing method were protected by patent claim No. P.435847 [67]. Table 1 shows the experimental design layouts of the study. The measurements of dependent variables were repeated at least five times.

The conducted experimental tests included direct measurements of the following variables: mass *m* [g] of the extrudate measuring lengths obtained in 15 s intervals, screw drive torque *M* [Nm], polymer melt pressure before the extrusion head *P* [MPa], and the total electric power supplied to the extruder *Q* [kW] (drawn by the drive, plasticizing, cooling and control system). The mass flow rate of the extruded plastic material *G* [g/s] and the specific energy consumption of the extruder *E_jc_* [J/g] were determined indirectly. The specific energy consumption *E_jc_* was defined as the ratio of the total electric power drawn by the extruder *Q* to the mass flow rate of the extruded material *G*. The following quantities characterizing the obtained blend were also determined: melt flow rate (*MFR*) [g/10 min] and density *ρ* [g/cm^3^].

The aforementioned *M*, *P*, *G*, *E_jc_*, *MFR*, *ρ* quantities were the dependent variables (subject to observation) adopted for the tests. The measurements conducted as per the adopted experimental design enabled the approximation of the relationships between dependent and independent variables, through a multivariable polynomial consisting of such terms as constant value, linear terms, square terms, and two-factor interaction term (Equation (1)). Where Y is the expected response value (Y stands for *M*, *P*, *G*, *MFR*, *ρ*, *E_jc_*), a_0_ is the constant value, while a_x_ are regression coefficients. The equation is as follows:

(1) $$Y\left(n.\;u\right)={\;a}_{0}\;+{\;a}_{1}n\;+{\;a}_{2}u\;+{\;a}_{3}n^{2}\;+{\;a}_{4}u^{2}\;+{\;a}_{12}nu$$

The properties of the obtained extrudate were also assessed. They included:

- Observing pellet and filler surfaces. This was performed with the use of an Eclipse LV100 ND optical microscope (Nikon, Tokyo, Japan), equipped with a DS-U3 camera and running on NIS-Elements AR 4.20.00 software (Nikon, Tokyo, Japan). The reflected light method was used for the observations. All samples were observed under identical magnification and illumination conditions, using the dark field technique.
- FTIR analysis of the samples. This was conducted using a TENSOR 27 FTIR spectrophotometer (Bruker, Billerica, MA, USA), equipped with an ATR (Attenuated Total Reflectance) attachment with a diamond crystal. The spectra (16 scans per spectrum) were collected for a range of 600–4000 cm^−1^ and a resolution of 4 cm^−1^.
- Differential scanning calorimetry (DSC) testing of the obtained pellets. This was undertaken using a 204 F1 Phoenix DSC scanning calorimeter (NETZSCH, Günzbung, Germany) and NETZSCH Proteus data processing software (NETZSCH, Günzbung, Germany), in accordance with standard ISO 11357-1:2016 [68]. DSC thermograms were recorded for the following cycles: heating (I) from −150 °C to 140 °C (at a rate of 10 K/min), cooling from 140 °C to −150 °C (at a rate of 10 K/min) and heating (II) from −150 °C to 140 °C (at a rate of 10 K/min). The samples, weighing approximately 10 mg, were tested in aluminum crucibles with a pierced lid. DSC curves were used to determine the degree of crystallinity *X_c_*, melting enthalpy Δ*H_m_*, melting point *T_m_*, crystallization temperature *T_c_* and glass transition temperature *T_g_* for the obtained composites. The degree of crystallinity was calculated from the relationship:
  $$X_{c}=\left(\frac{\Delta H}{\left(1-u\right)\times \Delta H_{100\%}}\right)\times 100\%$$
  assuming that for PBS, Δ*H*_100%_ = 110.3 J/g [69]. The DSC curve inflection point in the glass transition area was adopted as the glass transition temperature.
- Thermogravimetry (TG) testing of the extrudate in an oxidizing atmosphere. This was conducted with the use of an STA 449 F1 Jupiter thermal analyzer (NETZSCH, Günzbung, Germany) connected with a TENSOR 27 FTIR spectrophotometer (Bruker, Germany), which allowed simultaneous analysis of the gas products of the sample decomposition. The analyses were conducted in a temperature range of 40–800 °C, in an atmosphere of synthetic air with a gas flow rate of 25 mL/min. Samples, each weighing approximately 12 mg, were analyzed in Al_2_ O_3_ crucibles.
- Determining the melt flow rate (*MFR*) (150 °C/2.16 kg) in g/10 min. The measurement was taken using a MeltFlow TQ6841 extrusion plastometer by CEAST (Turin, Italy), based on the recommendations of ISO 1133-1:2011, method A [70].
- Testing standard density. This utilized the immersion method, as per standard ISO 1183-1 A [71]. The in-air sample mass was determined, and prior to establishing the in-water sample mass, it was held fully immersed in water for 24 h in order to achieve full imbibition. Filler standard density was determined via the pycnometric method.
- Determining the relationship between pressure *p*, specific volume *v* and temperature *T* during isobaric cooling. This occurred as per standard ISO 17744:2004 [72]. The measurements were conducted using a *p-v-T* 100 device from SWO Polymertechnik GmbH (Krefeld, Germany). The tested samples were heated up to a maximum measurement temperature of 165 °C and pressurized to a preset value (20, 50, 80 and 110 MPa). Next, the constant pressure was maintained and the samples were cooled down at a rate of 5 °C/min, down to a temperature of 35 °C, after which the process was repeated for the subsequent, higher pressure value.
- Establishing the moisture content of the obtained biocomposite pellets in wt.% according to standard EN ISO 585:1990 [73]. This resulted from measurement using a Radwag WPS 50 SX moisture analyzer (Radom, Poland). The pellets were first subjected to preliminary drying in a heating chamber at 80 °C for 48 h. The samples were then kept in an isolated room, with a humidity of 33% and a temperature of 20.8 °C. A moisture analyzer was used to measure the moisture of biocomposite pellets weighing 2.2–2.5 g (several dozen pellets), at a temperature of 120 °C. The measurements were taken after drying and after 1, 2, 5, 8 and 12 days, until all tested samples achieved equilibrium moisture content.

## 3. Results

The results of twin-screw extrusion of PBS/WB biocomposites are shown in Table 1. It contains the mean values of the studied dependent variables, *M*, *P*, *G*, *E_jc_*, *MFR*, *ρ*, as determined for individual experimental design layouts. Based on the collected measurement results, the authors estimated regression coefficients for empirical models expressing the cause-and-effect relationships appearing between dependent variables (observed) and the set of independent variables under preset process conditions. The experimental test results were statistically processed using variance analysis. The verification of empirical model structural correctness and a statistical evaluation of individual terms of determined regression equations were also conducted. The Pareto chart of standardized effects was used for the statistical evaluation of the impact of individual independent variables on the properties of the obtained extrudate. This enabled demonstrating an overview of the impact of individual regression equation terms on the value of a modeled dependent variable. Graphed standardized effect absolute values which exceeded the vertical line corresponding to the adopted significance level of *p* = 0.05 are deemed statistically significant.

### 3.1. Processing Characteristics

#### 3.1.1. Mass Flow Rate

**Figure 2:**
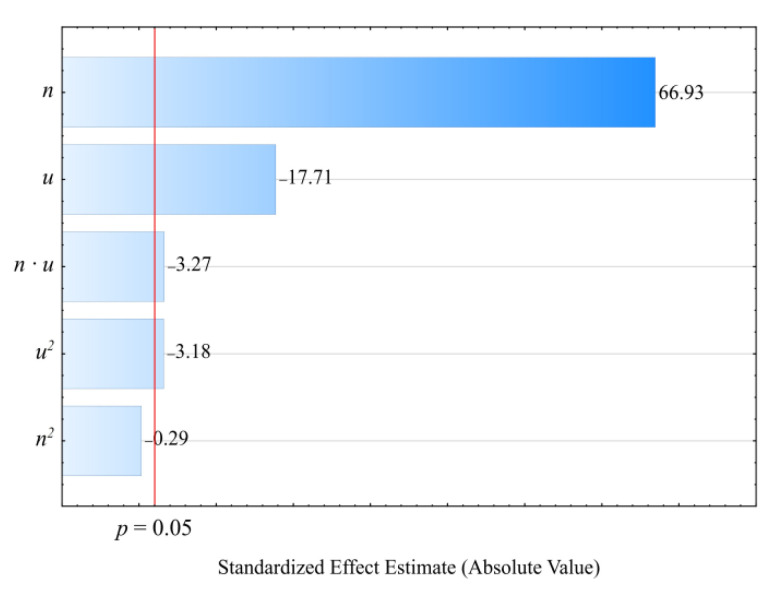
Pareto charts of the standardized effects for the empirical model *G*; the vertical line in the plot corresponds to the arbitrarily chosen level of significance (*p* = 0.05).

**Figure 3:**
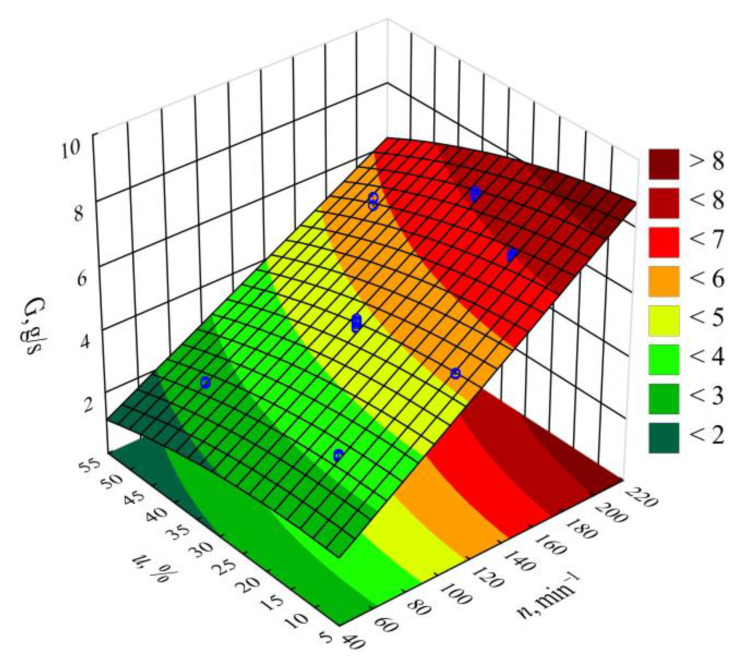
Response surface graph for the polymer mass flow rate *G* versus the screw rotational speed *n* and bran mass fraction *u.*

Figure 2 and Figure 3 show the results for modeling the mass flow rate of the extruded material *G*. The studies adopted an empirical model in the form of a polynomial (Equation (2)):

(2) $$G=\;0.60555+0.03736n+\;0.01907u\;-\;0.000560u^{2}-0.000159nu$$

The statistical analysis results are shown in Table 2. It was observed that the screw rotational speed *n* had the greatest impact on the mass flow rate of the extruded material, and that this relationship was of a linear nature. The highest increase in the *G* value obtained during the tests, amounting to 4.92 g/s (225%), was the outcome of increasing the screw speed from 50 to 200 min^−1^, with the same bran content of 30 wt.% (experimental design layouts 5 and 6). The bran content also had a statistically significant impact on the mass flow rate. It was, however, significantly lower than that of screw rotational speed and had a negative effect. The highest of the tested increases in the bran content, from 10% to 50% at a constant screw speed of 125 min^−1^, resulted in the mass flow rate decreasing by 1.61 g/s (31%). This was caused by the melt viscosity of the blend increasing together with increasing bran content. Other authors studying the rheology of PBS with natural fillers indicated a similar phenomenon [51,61,74]. However, the significant increase in the mass flow rate together with increasing rotational speed is undoubtedly associated with growing material feeding rate and transportation speed along the plasticizing system, but can also be partially related to the rheological properties. Previous work has demonstrated that both PBS and its biocomposites exhibit a non-Newtonian character with a tendency for shear thinning behavior, which has a positive impact on the material mass flow rate [61,74]. The statistical analysis also showed a significant negative impact of the interactions between the screw rotational speed and bran content on the mass flow rate. The probable cause is the increase in the shear stresses resulting in the thinning of soft WB particles due to the influence of intensive shear stress, as well as the fact that the melt viscosity of LCF polymer blends increases with decreasing filler particle size [61,75]. The statistical analysis also exhibited a significant interaction between screw rotational speed and bran content on the flow rate.

#### 3.1.2. Screw Drive Torque

The empirical model for the screw drive torque *M* determined as a function of variables *n*, *u* is shown through a polynomial (Equation (3)):

(3) $$M=\;84.66243+0.10900n-1.23011u\;-0.00062n^{2}+0.00488nu$$

**Figure 4:**
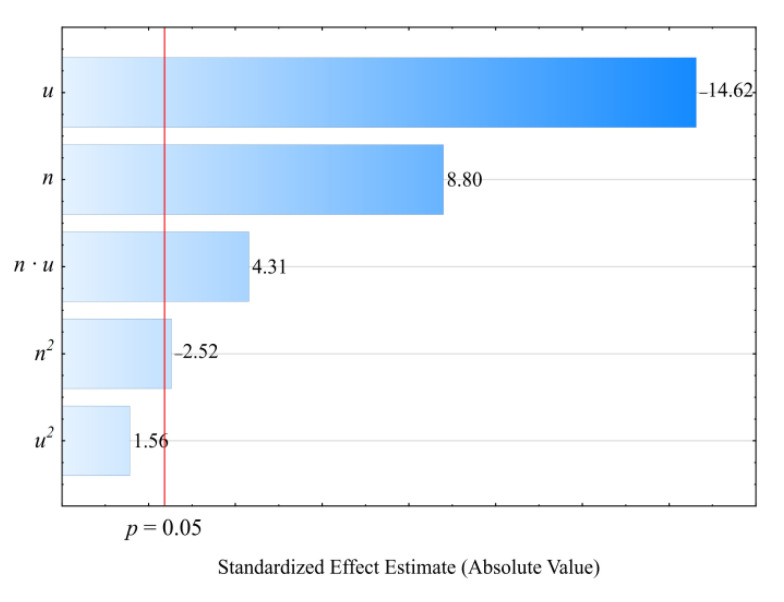
Pareto charts of the standardized effects for the empirical model *M* of the screw drive torque; the vertical line in the plot corresponds to the arbitrarily chosen level of significance (*p* = 0.05).

**Figure 5:**
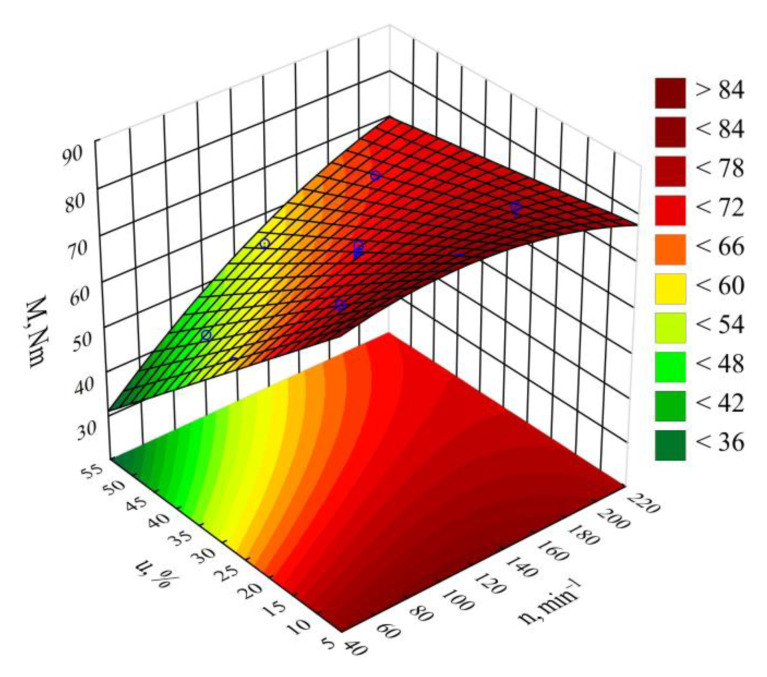
Response surface graph for the screw drive torque *M* versus the screw rotational speed *n* and bran mass fraction *u.*

The statistical analysis results regarding the adopted screw drive torque model are shown in Table 3. The mass content *u* of bran added to the blend has the highest impact on the torque value (Figure 4). Increasing bran content leads to decreasing the torque (Figure 5). Its greatest reduction, by 26 Nm (34%), was the outcome of increasing the bran content from 15.9% to 44.1%, and was observed between comparable (for the same screw speed) experimental design layouts 1 and 2. The source literature stipulates that despite the general increase in the blend melt viscosity, increasing LCF content also means that PBS shear thinning behavior intensifies with increasing filler content. This is explained by decreased activation energy, which stands for the amount of energy required to set a polymer molecule in motion against the frictional forces of adjacent macromolecules [61,74,76]. The increasing amount of gases, especially water vapor, released from bran during processing, resulting in the porosity of the processed blend, can significantly reduce the activation energy. On the other hand, increasing the screw rotational speed caused an increase in the screw drive torque. This is due to the simultaneous increase in the mass flow rate of the extruded blend because a gravity-fed hopper was used. Its highest increase by 18.8 Nm (37%) resulted from increasing the processing screw speed by 2.5-fold, and was observed between comparable experimental design layouts 2 and 4. Statistical analysis also showed the significant positive impact of the interactions between the screw rotational speed and bran content on the screw drive torque. This can be associated with the aforementioned thinning of WB particles.

#### 3.1.3. Pressure of Processed Blend

The determined relationships describing the processed blend pressure variability *P*, measured before the extrusion head, were shown through a polynomial (Equation (4)):

(4) $$P=\;1.49758+0.03603n+0.03124u\;-0.00009n^{2}+0.00062u^{2}+0.00018nu$$

**Figure 6:**
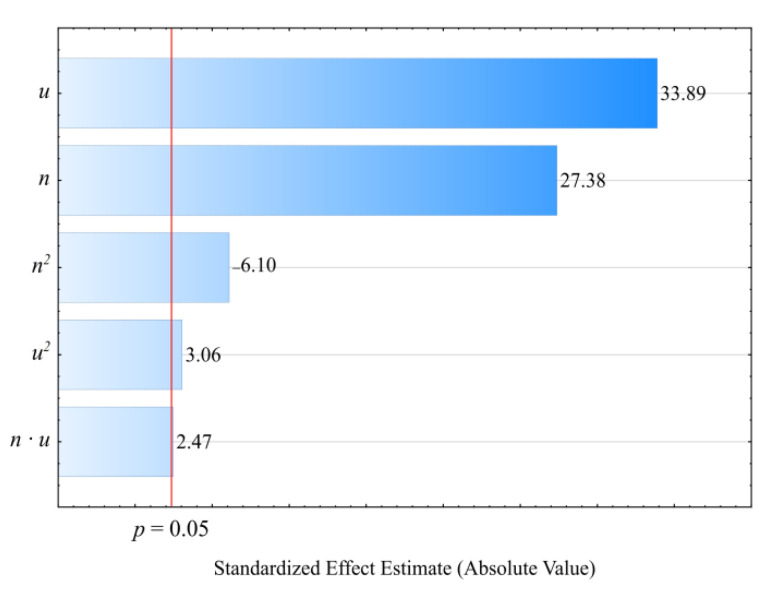
Pareto charts of the standardized effects for the empirical model *P* of the polymer blend pressure; the vertical line in the plot corresponds to the arbitrarily chosen level of significance (*p* = 0.05).

**Figure 7:**
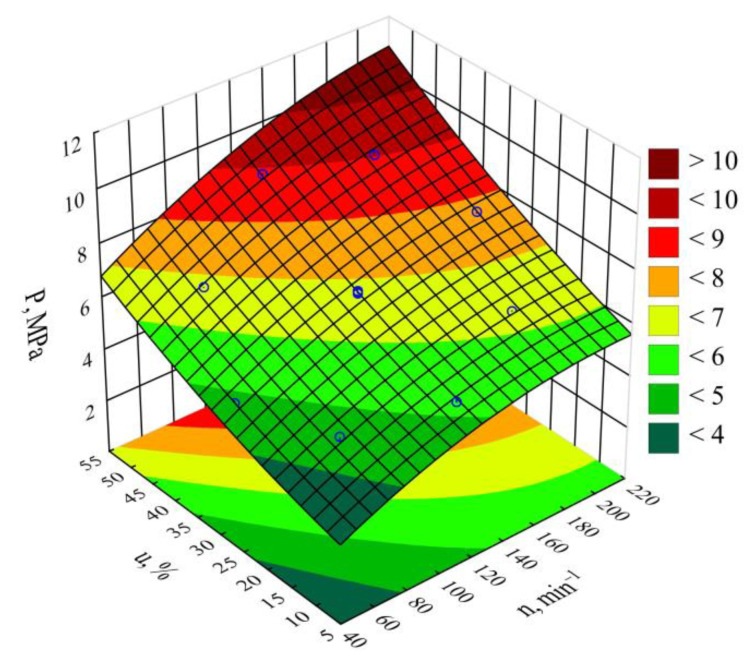
Response surface graph for the polymer blend pressure *P* versus the screw rotational speed *n* and bran mass fraction *u.*

Table 4 and Figure 6 reveal the outcome of a statistical analysis involving the impact of variable factors on pressure. Increasing both the screw rotational speed and the bran content resulted in a significantly higher processed blend pressure (Figure 7). This increase comes from enhancing both the mass flow rate of the extruded polymer blend (where a higher transport efficiency with the same cross-section of extrusion head channels results in an obvious increase in pressure) and its viscosity. The increase in the blend viscosity results from the shortened duration of its residence within a plasticizing system, as well as increasing bran content [51,61,74]. The highest pressure increase of 3.1 MPa (65%) is the end product of a fourfold increase in the screw speed. This was observed between comparable (with the same bran content of 30%) experimental design layouts 5 and 6. In contrast, reducing the bran content within the studied range from 10 wt.% to 50 wt.% (experimental design layouts 7 and 8) caused the pressure of the processed blend to increase by 3.8 MPa (75%). The modeling of the results also showed the presence of a statistically significant interaction between *n* and *u*, albeit with a much lower influence.

#### 3.1.4. Specific Energy Consumption

The obtained results of measuring the power consumed by the extruder and the mass flow rate of the extruded blend were used to calculate the specific energy consumption supplied to the extruder *E_jc_*. The empirical model describing the specific energy consumption was determined in the form of a polynomial (Equation (5)):

(5) $$E_{jc}=\;2221.4-13.027n+0.034n^{2}$$

**Figure 8:**
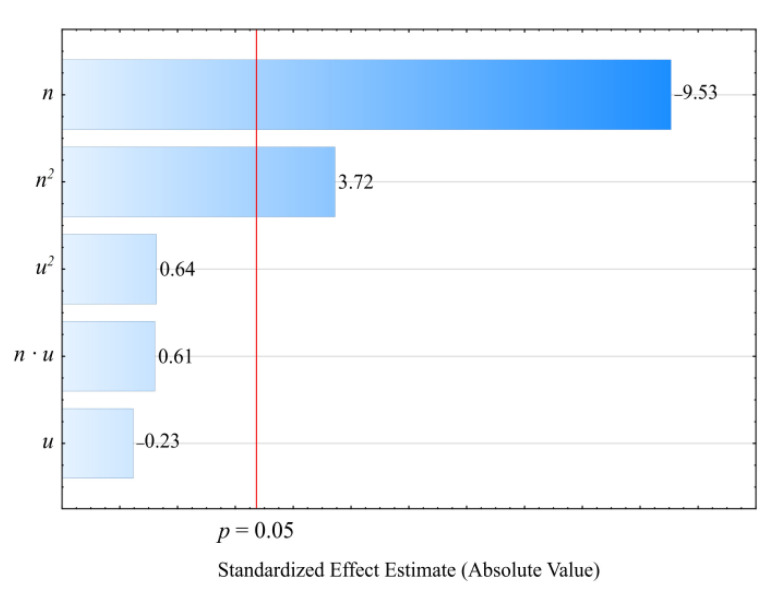
Pareto charts of the standardized effects for the empirical model of extruder’s specific energy consumption *E_jc_*; the vertical line in the plot corresponds to the arbitrarily chosen level of significance (*p* = 0.05).

**Figure 9:**
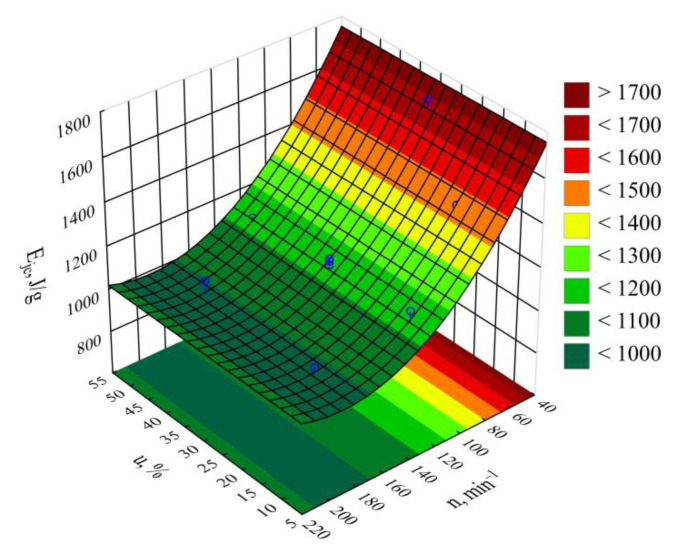
Response surface graph for the extruder’s specific energy consumption *E_jc_* versus the screw rotational speed *n* and bran mass fraction *u.*

The statistical analysis involving the impact of the studied extrusion conditions on the specific energy consumption showed that extruder screw rotational speed *n* had a statistically significant impact (Figure 8). The statistical analysis results regarding the adopted model *E_jc_* are shown in Table 5. Increasing the screw rotational speed resulted in a reduction in specific energy consumption. A fourfold increase in the processing screw rotational speed, from 50 min^−1^ to 200 min^−1^, resulted in a recorded decrease in the *E_jc_* value by 771 J/g (45%). This is due to the increase in the flow rate of the extruded material prevailing over the increase in the flux of energy supplied to the extruder. It also results from an increase in the shear stresses, together with the screw rotational speed, which leads to additional overheating of the blend due to internal friction. That PBS and LFC temperatures increase with the increase in the screw rotational speed was demonstrated in the source literature using a torque rheometer [61]. The thus-obtained autothermal effect enables spontaneous maintenance of the set temperature within the plasticizing system cylinder wall, and, hence, less frequent activation of the heaters in all nine heating zones. Therefore, in the case of high rotational speed values, due to the intensity of the autothermal phenomenon, the temperature must be lowered to a set level, and this is achieved by a pump forcing liquid circulation in the cooling system, hence, the inhibition of further decrease in the specific energy consumption. On the other hand, no significant impact within the set bran content range on the specific energy consumption was found. Figure 9 shows the response surface for the specific energy consumption depending on the aforementioned factors, as obtained through modeling.

### 3.2. Microstructure

**Figure 10:**
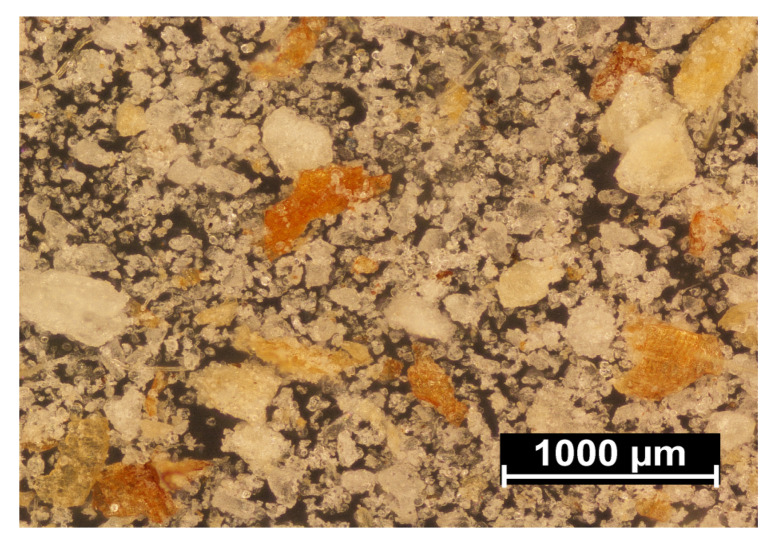
Microscopic image of the applied filler.

Figure 10 shows a microphotograph of the used filler in the form of wheat bran, after separating the fractions on a sieve with a mesh size of 0.2 mm. The image shows brown or light-brown flakes, similar in size to the upper range or smaller, formed as a result of their thinning. These are the actual bran, i.e., a water- and oxygen-permeable cellulose- and lignin-rich wheat grain husk. Significant amounts of smaller structures, in the form of white and grey crystals, were also observed. These are fragments of the aleurone layer, which is the outermost endosperm layer, containing mainly proteins and fats, as well as the middle endosperm, which primarily consists of starch. The degree of bran contamination with endosperm particles depends mainly on the grain grinding method, the type of wheat and the brittleness of grain outer layers, i.e., bran and the aleurone layer. Hard cultivars of wheat contain more brittle bran, which tend to chip off in small pieces together with endosperm fragments, during grinding. In the grinding of soft cultivars of wheat, the bran is more flexible and comes off in the form of large flakes, but it is also more challenging to grind into fine powder. Thus, wheat bran composition can significantly differ [77,78,79,80].

**Figure 11:**
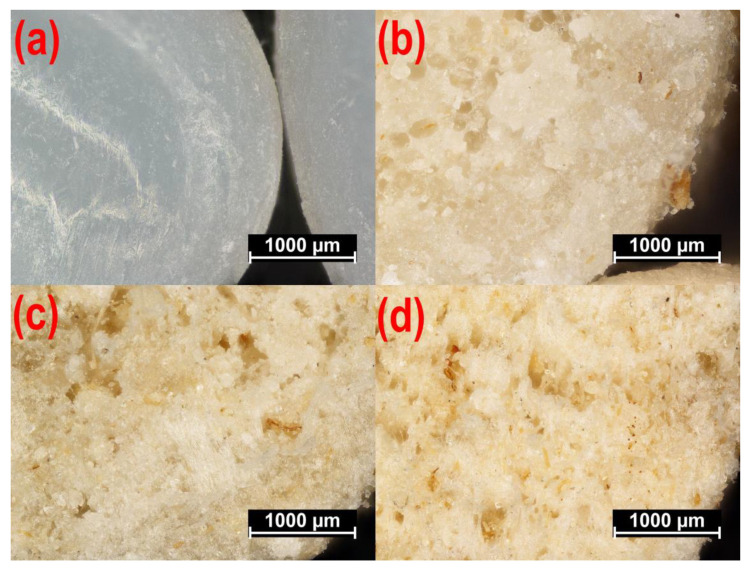
The microscopic images of non-processed poly (butylene succinate) (PBS) (**a**), and the fractures of composite pellets obtained for a screw rotational speed of n = 125 min^−1^ and a bran content of (**b**) *u* = 10 wt.%, (**c**) *u* = 30 wt.%, (**d**) *u* = 50 wt.%.

Microscopic images of PBS pellets and composite pellets obtained at a screw rotational speed of 125 min^−1^ are shown in Figure 11. The observed cross-section of PBS pellets provided by the manufacturer (Figure 11a) does not indicate any defects, with the pellets being white. The photos showing pellets with a bran content of 10 wt.% (Figure 11b), 30 wt.% (Figure 11c) and 50 wt.% (Figure 11d), however, indicate numerous defects in the form of pores. The cause behind their formation was the presence of structurally-bound water in the filler particles. This expanded after leaving the extrusion head and meeting the reduction in atmospheric pressure. In this situation, the greater the WB content, the higher the pore quantity. Figure 11b shows individual pores of regular shape, located solely near the pellet center. This is associated with the small amount of bran and rapid crystallization of the outer material layer when cooled within the water bath. In the case of higher WB content (Figure 11c,d), the pore count increases, and these interconnect into larger, irregular voids that appear near the pellet edge. Still, a uniform distribution of the filler was found in all cases, and WB particles were observed both within the material mass as well as on the surface of the biocomposite pellet. The color of the blend changes with increased WB content.

### 3.3. FTIR

**Figure 12:**
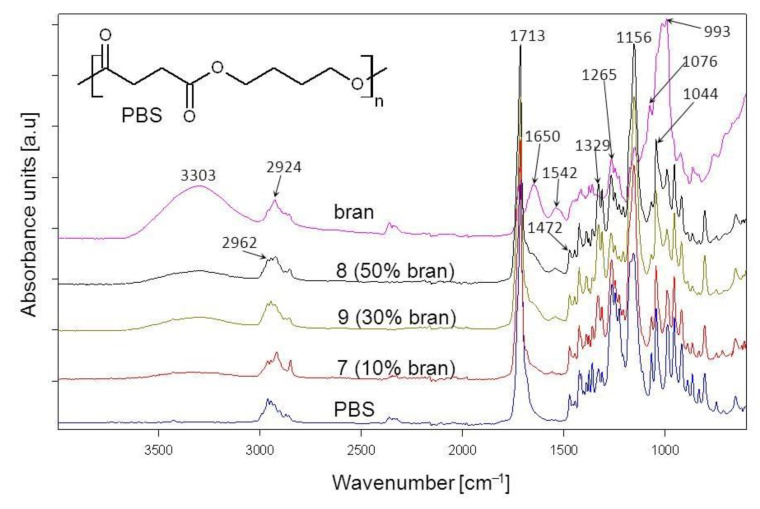
ATR-FTIR spectra of PBS, bran and their composites obtained pursuant to research plan system 7, 8, 9 with different bran content.

The chemical structure of the obtained biocomposites was confirmed through ATR-FTIR analysis. The spectrum of neat PBS (Figure 12) contains visible absorption bands that are characteristic for substances with an ester group within their chemical structure: an absorption band at 1713 cm^−1^ that comes from the vibrations of the –C=O group, as well as bands at 1265 cm^−1^, 1156 cm^−1^ and 1044 cm^−1^ that correspond to the vibrations of the –C-O-C– and –O-(C=O) groups [81]. In addition, absorption bands at 2962–2857 cm^−1^, 1472 cm^−1^ and 1329 cm^−1^ come from the vibrations of aliphatic methylene groups [74].

The wheat bran spectrum (Figure 12) contains absorption bands characteristic for polysaccharides, phenolic and lipid compounds, proteins and water. Vibrations of –OH groups (visible in polysaccharides, phenolic compounds and water) are visible on the spectrum as a wide absorption band at 3303 cm^−1^, whereas absorption bands at 2924 cm^−1^ and 2854 cm^−1^ result from the vibrations of methyl and methylene groups. The absorption band at 1716 cm^−1^ is due to vibrations of the –C=O groups present in carbonyl compounds contained in pectins and hemicellulose [63,82,83]. The broadband at 1649 cm^−1^ results from the vibrations of the –OH group from water as absorbed by starch [84,85]. Furthermore, absorption bands at 1650 and 1542 cm^−1^, respectively, confirm the presence of protein C-N and N-H amide groups [86]. Moreover, absorption bands at 1151 cm^−1^, 1076 cm^−1^, 1017 cm^−1^ and 993 cm^−1^ result from the vibrations of the –C-O-C– and –C-O– groups found in polysaccharides [64]. Figure 12 also shows sample spectra of biocomposites obtained with the use of 10%, 30% and 50% of bran. It can be seen that the absorption bands present on these spectra overlap with the bands on the original component spectra, with the only change being the intensity of individual spectra, which depends on the quantitative composition of the original mixture. No new absorption bands can be observed on the biocomposite spectrum, which indicates that in the course of their formation, no chemical bonds between the components were created, and they were only physically mixed. The chemical interaction that can be expected within a composite structure, given the presence of ester groups in PBS and hydroxyl groups in bran, is through hydrogen bonding. This could be evidenced by the slight shift of the –C=O group absorption band. However, due to the presence of carbonyl groups in bran, which overlap on the spectrum with –C=O groups in PBS, it is hard to unequivocally state, based on FTIR spectra, the existence of hydrogen interactions within the structure of the biocomposites in question.

### 3.4. DSC

**Figure 13:**
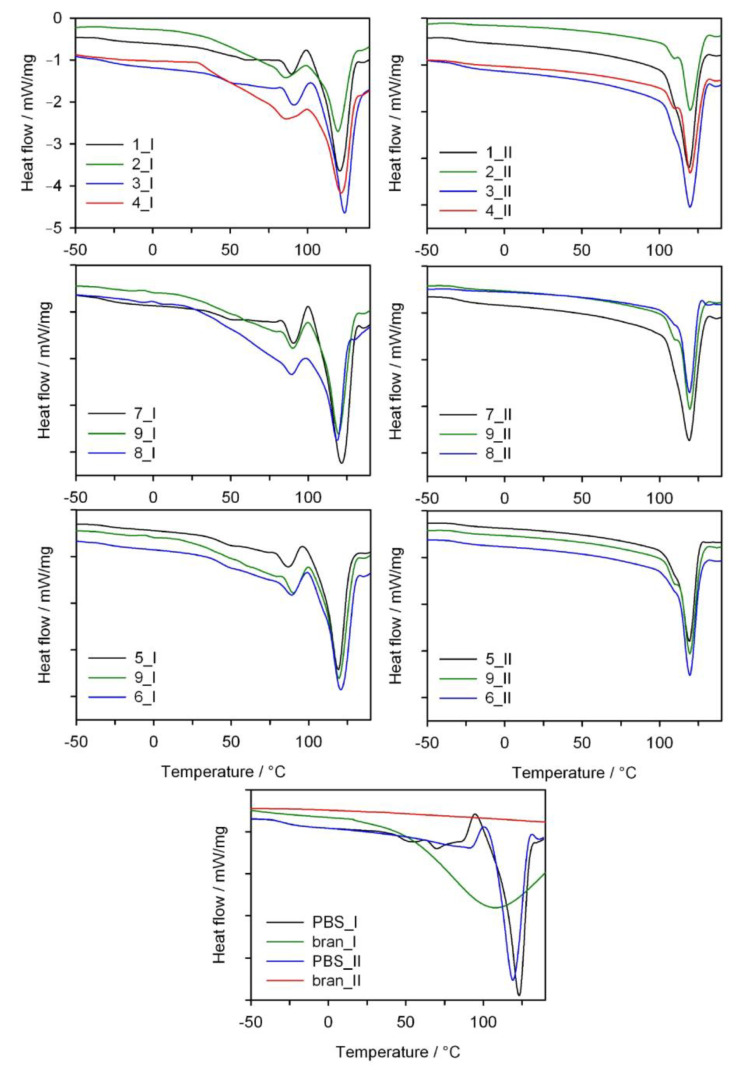
DSC thermograms of the first (I) and second (II) heating scans of neat PBS and its composites with bran (exo up).

The thermal properties of PBS/WB composites were determined based on DSC measurements conducted in the heating (I), cooling and heating (II) cycle, with the results shown in Figure 13. Table 6 shows the value of melting points *T_m_*, crystallization *T_c_* and glass transition temperature *T_g_*, the enthalpy of melting Δ*H_m_* and the degree of crystallinity *X_c_* of the tested composite materials used for their production, i.e., PBS and bran. The glass transition temperatures of the biocomposites and neat PBS are similar and fall within a range from −29 °C to −31 °C. No significant impact of the amount of bran and the screw rotational speed on the *T_g_* value was observed, which suggests that PBS secondary chain relaxation is similar in all composites. In the case of DSC thermograms from the first heating cycle for all composites, we can observe a significant endothermal peak at approximately 120 °C. This is associated with the PBS melting process. Analyzing the *T_m_* value shows that, compared to neat PBS, adding bran reduces the melting point of the composites. Furthermore, DSC thermograms from the first heating cycle show a minor separate endothermal peak, with its maximum at approximately 89 °C, which fades or shifts towards higher temperatures during the second heating cycle. The presence of this peak can be linked to the evaporation process of water absorbed within the bran structure (note: the bran DCS curve shows a broad endothermal peak related to water desorption). The conducted thermogravimetric analysis also shows a mass loss at a temperature of up to approximately 110 °C, which can be associated with the evaporation of water. However, this minor endothermal peak is narrow and distinct. This suggests it originates from the melting process rather than water evaporation. The presence of an additional peak on the DCS curves that originates from the melting process was also reported for PBS composites with palm fibers [87], PBS with rice straw [74], PBS with hemp fibers and with hemp shives [46], as well as PBS blends with polylactide and poly(3-hydroxybutyrate-*co*-hydroxyvalerate) [88] and even for neat PBS [89]. As was stipulated in these reports, this first endothermic peak can result from the melting of less-perfect crystals of PBS. The thermograms for the first heating cycle (Figure 13) also indicate that the higher the bran fraction content in a composite, the more the first endothermal peak is shifted towards a lower temperature. This suggests that higher bran content in the course of composite production can lead to the formation of smaller PBS crystallites. Therefore, bran can act as a crystallization promoter while simultaneously restricting the mobility of polymer chains, which results in the formation of crystallites of various sizes [90]. Re-cooling and then re-heating the composites resulted in the unification of the crystalline structure, and in consequence, the DCS curves for the second heating cycle contain only a minor arm on the main endothermal peak that is more distinct for samples with a higher bran content. Of note, two peaks associated with the polymer melting process are still observed in the case of neat PBS.

The enthalpy of melting Δ*H_m_* from the first heating scan was determined by taking the high melting peak into account. A general conclusion is that the increased bran content in the composites resulted in a reduced Δ*H_m_* value and degree of crystallinity compared to neat PBS, both for the first and second heating cycles. The initial *X_c_* value for PBS is 71.4% (II heating scan) and decreases down to 48.4% for a 50% bran content. The presence of bran probably interferes with the PBS chain organization process and leads to a reduced biocomposite degree of crystallinity. In contrast, increased screw rotational speed, with constant bran content, leads to increased Δ*H_m_* and *X_c_* of the composites (experimental design layouts 5, 9, 6 and 1, 3 and 2, 4). This fact can be explained by a more homogeneous distribution of bran within a PBS matrix and its thinning through intensive shearing during processing, which resulted in obtaining more regular crystallite shapes. When considering the crystallization temperature (*T_c_*) of neat PBS and composites, it can be seen that adding bran shifts the *T_c_* value from 78.4 °C towards a higher temperature, until 87.5 °C for experimental design layout 7, which contains 10% bran, and then decreases with increasing bran content. This may suggest that, in the case of a lower bran content, WB is more uniformly distributed within the PBS matrix and can act as nucleation center during the PBS crystallization process.

### 3.5. Thermal Resistance

**Figure 14:**
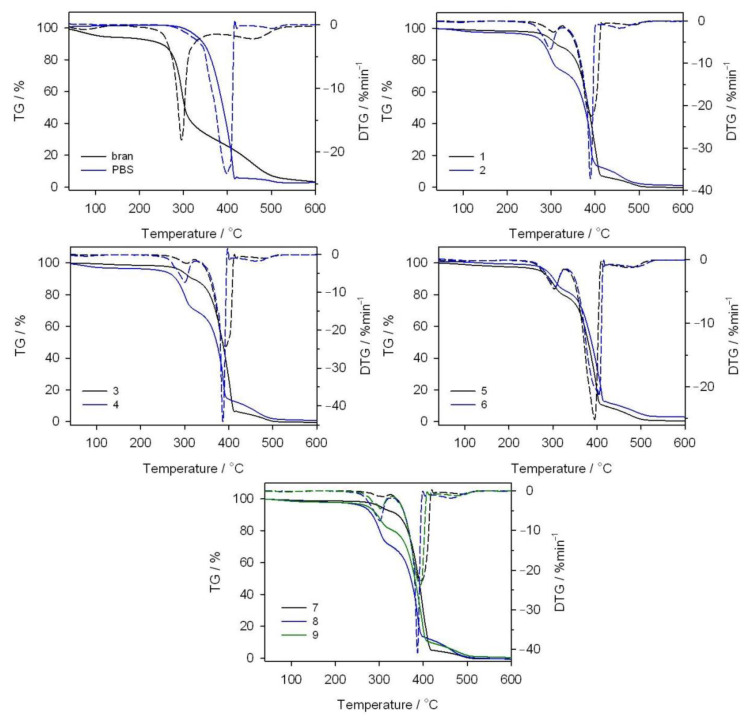
TG and DTG curves obtained in an oxidizing atmosphere for bran, PBS and their composites.

**Figure 15:**
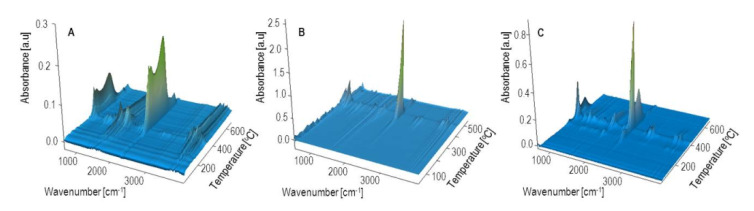
Three dimensional FTIR diagrams of gaseous decomposition products of bran (**A**) PBS (**B**) and biocomposite 2 (**C**) obtained with 44% of bran.

**Figure 16:**
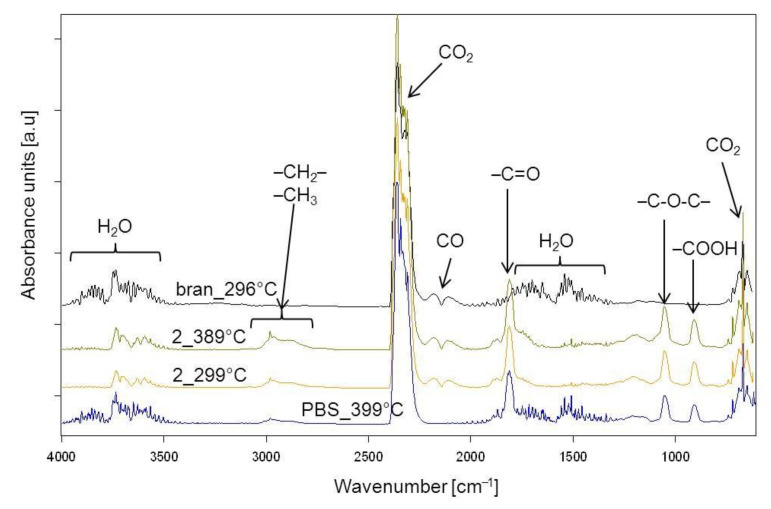
Extracted at the maximum value of the FTIR spectra for gaseous decomposition products of PBS, bran and biocomposite 2 obtained with 44% bran.

The thermal resistance of the obtained PBS/WB composites, in a synthetic air atmosphere, was studied through thermogravimetric analysis. The tests also involved analyzing gaseous composite decomposition products. Figure 14 shows the TG and derivative thermogravimetry (DTG) curves for the tested materials, while Table 7 contains basic parameters characterizing their thermal resistance. The PBS TG curve shows that it decomposes in one major step, with the maximum at 399 °C and a mass loss of 94.3%. Based on the FTIR spectrum for gaseous decomposition products (Figure 15 and Figure 16), one can conclude that this decomposition starts with ester bond hydrolysis, which leads to the formation of succinic acid and 1,4-butanediol [91,92], followed by an immediate oxidation process. Such a course of thermal degradation is evidenced by absorption bands at 2981–2897 cm^−1^ (vibrations of the –CH_2_– and –CH_3_ groups present in succinic acid and 1,4-butanediol), at 1811 cm^−1^ (–C=O present in succinic acid), 1053 cm^−1^ (–C-O-C– in succinic acid and 1,4-butanediol), 909 cm^−1^ (–COOH in succinic acid), as well as intensive absorption bands at 2359–2310 cm^−1^ and 669 cm^−1^ originating from carbon dioxide and at approximately 4000–3500 cm^−1^ and 1800–1300 cm^−1^ originating from water.

The DTG curve peak, with its maximum at 502 °C and a negligible mass loss, comes from the ultimate decomposition of the deposit formed at the main stage of PBS decomposition. Only CO_2_ and water are emitted (Figure 15). The TG and DTG curves for bran (Figure 14) indicate that thermal degradation is preceded by the evaporation of absorbed water (6% mass loss), at a temperature of up to 120 °C [56]. The main bran decomposition stage is most rapid at 296 °C. The FTIR spectrum for gaseous decomposition products at this temperature contains mainly absorption bands from water and carbon dioxide and monoxide. This means that the decomposition follows the oxidation mechanism.

Given the TG and DTG curves for biocomposites, it can be noticed that their thermal decomposition is divided into three stages, which are most rapid at temperatures of approximately 300 °C, 400 °C and 480 °C. The mass loss value Δ*m_1_* depends on the bran content in the blend and increases together with increasing bran content, reaching a maximum of almost 30% for experimental design layout 8. The T_max1_ value shifts with the bran content, from 308 °C for 10% bran in experimental design layout 7, to 299 °C for 50% bran in layout 8. Based on the FTIR spectrum for gaseous decomposition products for layout 2, it can be concluded that both bran and PBS began to decompose at this stage. This is evidenced by the distinct absorption bands at 1811 cm^−1^, 1053 cm^−1^ and 909 cm^−1^. The next stage of biocomposite decomposition, at approximately 400 °C, involves further PBS degradation that is associated with ester bond hydrolysis and parallel oxidation. Carbon monoxide is also emitted (absorption bands at 2181 and 2114 cm^−1^) at this stage, unlike the situation of neat PBS (Figure 16). The last decomposition stage, at approximately 480 °C and with the least mass loss, is caused by the oxidation of deposits formed in the course of the previous stages, which leads to carbon dioxide and water emissions (Figure 15).

Conclusions in respect of the composite thermal resistance can be drawn from the *T_5%_* parameter, determined at a 5% sample mass loss. Table 7 shows that, compared to PBS-bran composites, neat PBS is characterized by a higher thermal resistance. Hence, increasing the bran content in composites leads to the deterioration of their thermal resistance, which reaches its maximum at 260 °C for a sample with a 50% bran content. The *T_50%_* value changes in a similar manner. Therefore, the lower thermal resistance of bran, relative to PBS, determines the thermal resistance of biocomposites.

The thermal resistance of biocomposites can also be analyzed by taking into account the screw rotational speed (*n*), changed in the range of 50–200 min^−1^. When comparing the pairs of experimental design layouts 1, 3 and 2, 4 obtained with 15.9% and 44.1% bran, with the *n* equal to 72 and 178 min^−1^, and layouts 5, 9 and 6 obtained with 30% bran and *n* equal to 50, 125 and 200 min^−1^, respectively, it can be seen that the screw rotational speed for each of these cases caused a reduction in the composite thermal resistance, manifested in the lowered *T_5%_* and *T_50%_* values (Table 7). It can be assumed that higher screw rotational speed within the extrusion process results in better component homogenization and, in consequence, a more homogeneous dispersion of bran within the PBS matrix of the end product. Because bran content limits the thermal resistance of composites, their uniform distribution within a composite structure accelerates its thermal decomposition. The mechanisms behind thermomechanical and thermo-oxidative chain breaking can also be a potential cause. In combination with the presence of moisture, they will lead to reduced molecular weight of the macromolecules. This was demonstrated through the repeated processing and prolonged heating of PBS, especially under the presence of the autothermal effect, which may cause the local material temperature to be higher than the set cylinder wall temperature [60,93,94].

### 3.6. MFR

**Figure 17:**
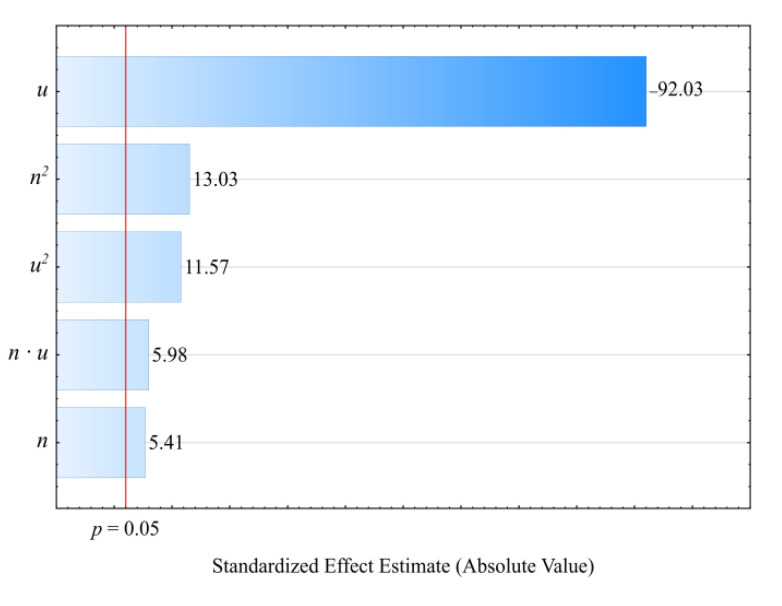
Pareto charts of the standardized effects for the empirical model of *MFR*; the vertical line in the plot corresponds to the arbitrarily chosen level of significance (*p* = 0.05).

**Figure 18:**
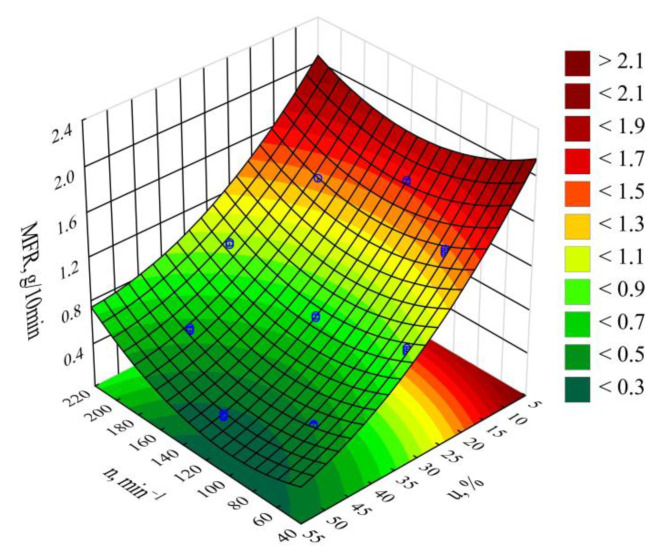
*MFR* response surface graph depending on the wheat bran content *u* and screw rotational speed *n.*

The modeling results for the melt flow rate (*MFR*) are shown in Figure 17 and Figure 18. An empirical model in the form of a polynomial was adopted. This also includes, besides linear and square terms, a binary interaction term (Equation (6)):

(6) $$\rho \;=\;2.813219-0.010452n-0.066258u+0.000037n^{2}+0.000465u^{2}+0.000055nu$$

The statistical analysis results are shown in Table 8. Modeling the melt flow rate (*MFR*) indicated that its value was most impacted by wheat bran content in the blend. *MFR* values were seen to decrease with increasing bran content. The highest *MFR* decrease observed during the tests, amounting to 1.28 g/10 min (83%), resulted from increasing the bran mass content from 10% to 50%, while maintaining a constant screw rotational speed (experimental design layouts 7 and 9). The screw rotational speed, and thus, the processed blend shear rate, has a significantly smaller impact on the *MFR* value. The observed changes resulting from increasing the screw speed within the studied range did not exceed 0.18 g/10 min. These can be caused by changes in the structure of the processed blend as a result of increasing shear stresses. The influence of the interaction between bran and screw speed is also noticeable. It should be noted that the flow rate for all tested blends was significantly lower relative to sole poly(butylene succinate), for which the measured *MFR* value was 2.05 ± 0.01 g/10 min. Introducing a dispersed filler into PBS resulted in an increase in the blend melt viscosity that was manifested by decreased values of the melt flow rate. A similar impact of the plant filler content on the melt flow rate (*MFR*) value was shown in other works that reviewed blends of PBS with natural origin fillers [51,61,74,76]. Moreover, the much greater influence of bran content on *MFR* than on the mass flow rate suggests that shear occurring during screw plasticizing has a significant impact on the processability of the studied blends.

### 3.7. Density

**Figure 19:**
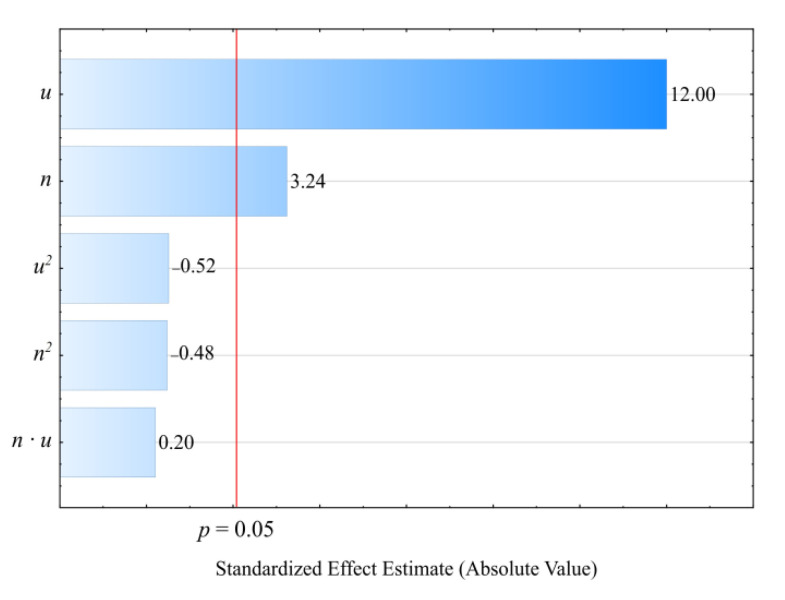
Pareto charts of the standardized effects for the empirical model density *ρ*; the vertical line in the plot corresponds to the arbitrarily chosen level of significance (*p* = 0.05).

Based on the conducted studies, it was concluded that the density *ρ* of the extruded blend is significantly impacted by both the mass content of the bran therein as well as the extruder screw rotational speed. Statistical analyses showed only the statistically significant influence of the linear terms of the model equation (Figure 19). This is why a polynomial taking into account only these terms and the constant value was applied for modeling (Equation (7)):

(7) ρ = 1.031357+0.000324n+0.004496u

**Figure 20:**
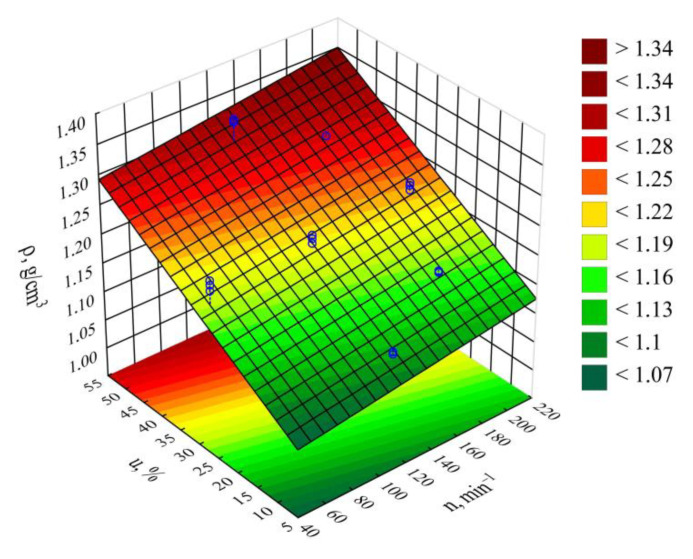
Response surface graph for the density *ρ* versus the screw rotational speed *n* and bran content *u.*

The statistical analysis results are shown in Table 9. Blend density modeling (Figure 20) showed that its value was most impacted by the bran mass content (Figure 19). As this content increases, a proportional increase in the extruded polymer blend density was observed. The highest density increase seen during the tests, amounting to 0.219 g^3^/cm (19.7%), resulted from increasing the bran mass content from 10% to 50%, while maintaining a constant extruder screw rotational speed of 125 min^−1^ (experimental design layouts 7 and 8).

It should be noted that blend density demonstrated lower values than that expected from the densities of its constituents. Namely, the measured poly(butylene succinate) density is *ρ* = 1.2460 ± 0.0099 g/cm^3^, while the density of wheat bran is ρ = 1.5347 ± 0.0084 g/cm^3^. Blend density values lower than expected are caused by the significant degree of porosity of its extrudate samples, which is clearly evident on the microscopic images (Figure 11b–d) which contain pores, the number and size of which increase with bran content. These are largely the result of moisture release from the bran, which comes in the form of water vapor, and partially from the blend ingredient thermal decomposition products. When determining the blend density using the immersion method, prior to determining the mass in water, the samples were held in distilled water for 24 h in order to achieve full soaking. In doing this, while the water probably penetrated the tested pellets throughout the entire volume, gas could have remained inside the pores, since it takes much longer to permeate gas on the outside. This is why the obtained measurement results should be treated as the apparent density of the tested blend.

The impact of extruder screw rotational speed on blend composition is many times lower than that of bran content. Its increase resulted in a slight, but statistically significant proportional increase in density. The highest density increase observed during the tests, amounting to 0.018 g/cm^3^ (1.5%), resulted from increasing the screw speed from 50 min^−1^ to 200 min^−1^, while maintaining a constant bran content of 30% (experimental design layouts 5 and 6).

### 3.8. p-v-T Graphs

**Figure 21:**
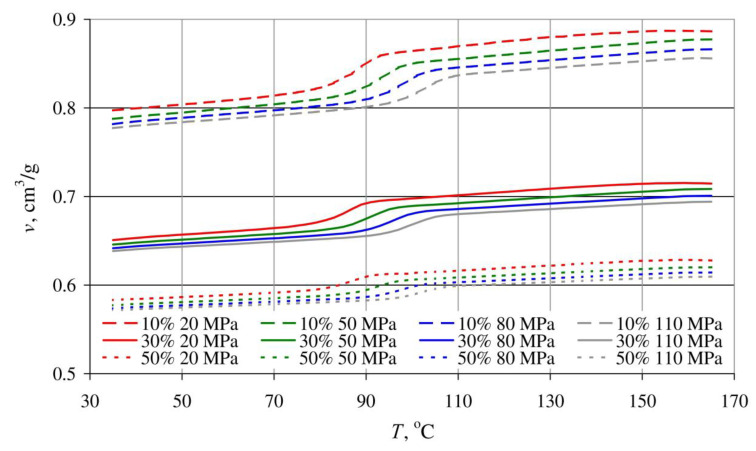
Dependence of the specific volume *v* on temperature *T* of blends obtained with the same extruder screw rotational speed of 125 min^−1^ and different bran contents: 10%—dashed lines, 30%—solid lines, 50%—dotted lines, for various pressure values.

**Figure 22:**
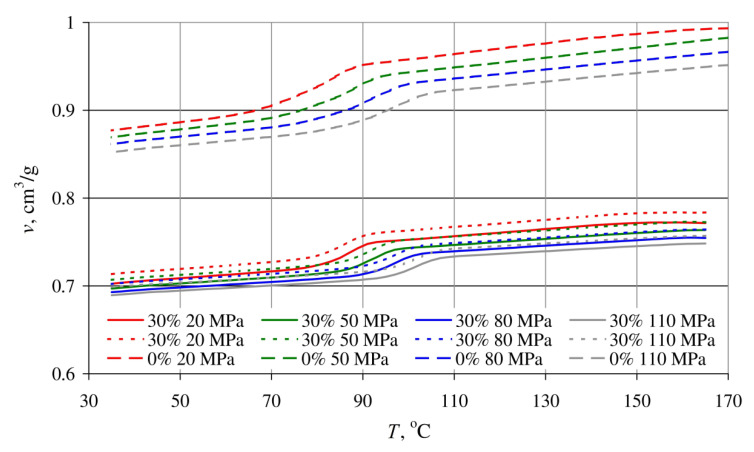
Dependence of the specific volume *v* on temperature *T* of a blend with a bran mass content of 30%, obtained for extruder screw rotational speeds of 50 min^−1^—solid lines and 200 min^−1^—dotted lines, and poly(butylene succinate) without added bran—dashed lines, for various pressure values.

The results of tests covering the relationship between pressure *p*, specific volume *v* and temperature *T* in the course of isobaric cooling of the tested blends are shown in Figure 21 and Figure 22.

The *p-v-T* graphs in Figure 21 are of the results for blends obtained with a constant extruder screw rotational speed of 125 min^−1^, differing in terms of bran content, namely 10%, 30% and 50%. Figure 22 shows the measurement results for blends with a bran content of 30%, obtained with extreme screw rotational speeds of 50 min^−1^ and 200 min^−1^. For comparative purposes, Figure 22 also shows the results of tests involving poly(butylene succinate) without added bran.

The shape of all obtained curves is very similar, but it can also be seen that the curve inflections shift towards higher temperature values with increasing pressure. This indicates that increasing crystallization temperature and the resulting rapid decrease in the specific volume are responsible for the phase transition.

The decrease in specific volume along with increased bran content is clearly visible. The lowest specific volume, 0.571 cm^3^/g, was exhibited by the blend with the highest (50%) content of wheat bran (at a temperature of 35 °C and pressure of 110 MPa). Under the same conditions, the specific volume of samples with a 30% bran content amounted to 0.638 cm^3^/g, 0.777 cm^3^/g for a 10% bran content, and 0.852 cm^3^/g for neat poly (butylene succinate).

Increased bran content also results in a significantly lower decrease in the specific volume in the phase transition area and the fact that the phase transition occurs over a wider temperature range. This is indicated by it becoming less and less distinct. These changes deepen with increasing pressure. A reduction in the blend specific volume, at a pressure of 20 MPa due to cooling down from 165 °C to 35 °C, amounted to 0.089 cm^3^/g with a 10% bran content (10% reduction), and 0.064 cm^3^/g for a 30% bran content (9% reduction). In the case of a 110 MPa pressure, the specific volume reduction was 0.079 cm^3^/g (9% reduction—10% bran content) and 0.056 cm^3^/g (8% reduction—30% brand content), respectively, as well as 0.039 cm^3^/g (6% reduction—50% bran content). The intensity of specific volume changes of neat PBS with decreasing temperature is similar, and it amounts to 0.116 cm^3^/g (11.6%) for a pressure of 20 MPa and 0.099 cm^3^/g (10.4%) for a pressure of 110 MPa. The aforementioned description can, therefore, be interpreted as a growing dimensional stability of the blend with increasing wheat bran content during cooling and pressure reduction.

On the other hand, the processing screw rotational speed over the tested range did not show significant impact on the determined *p-v-T* relationships. The curves obtained for the lowest 50 min^−1^ and highest 200 min^−1^ screw speeds are also identical (Figure 22), with minor differences in terms of the measurement errors.

### 3.9. Moisture Absorbance

**Figure 23:**
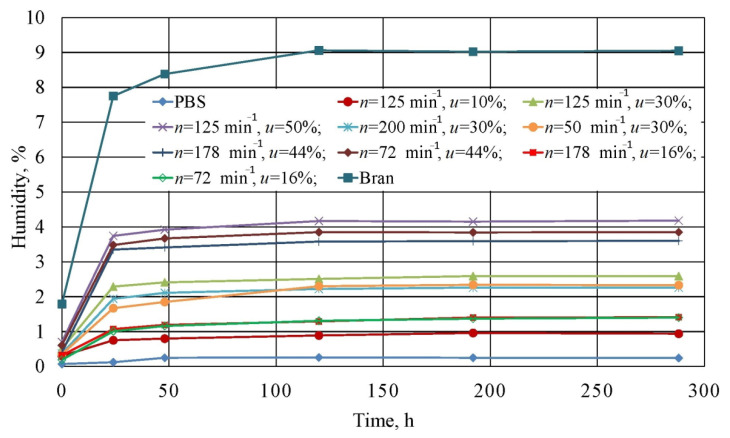
Relationship between the moisture absorbed by the tested blends, PBS and wheat bran itself from air and the exposure time.

The measurement results for the moisture absorbance of the studied composite blends, non-modified PBS, and wheat bran are shown in Figure 23.

The greatest moisture absorbance among all of the samples was exhibited by bran itself. Figure 23 shows that they were characterized by a high moisture of 1.79 wt.% directly after drying, and equal to 7.75 wt.% after 24 h, while an equilibrium moisture content of 9.05 wt.% was achieved by bran after 120 h. Such a high moisture absorbance is determined by the filler’s composition and the structure of its macromolecules, which contain various functional groups able to retain water. The water retention capacity of wheat bran varies in the range of 4–4.8 g of water/g, and can increase up to 2–3-fold in the case of very high thinning [77,95,96]. This explains the high moisture values that were obtained in conditions of relatively low air humidity.

In contrast, unmodified PBS was characterized by poor moisture absorbance. Here, water content was equal to 0.07 wt.% after drying, which means it was within the range of 0.07–0.1 wt.% that is recommended for processing [16,62]. After 24 h, the figure amounted to 0.12 wt.%, and after 48 h, equilibrium moisture at a level of 0.25 wt.% was achieved. This indicates that PBS pellets should be processed up to 24 h after drying.

The moisture absorbance of the studied blends increased with increasing bran content. Analogous moisture test results for PBS with lignocellulosic fillers can be found in scientific literature [97,98]. Directly after drying, all tested experimental design layouts 1 to 9 exhibited higher moisture values than that recommended for PBS processing. These ranged from 0.3 wt.% (*u* = 10%) to 0.7 wt.% (*u* = 50%). The highest moisture increase occurred within the first 24 h, and then significantly slowed down. Differences in the time to achieve equilibrium moisture content depended on bran content. Experimental design layouts with a bran content of u = 10% and u = 16% achieved equilibrium after 8 days, while other blends achieved this in 5 days. This is most likely due to the decreasing distances between bran particles in the material volume. The highest moisture value was recorded for experimental design layout 8, and amounted to 4.17%. No significant impact of the rotational speed on moisture absorbance was observed.

Furthermore, our work showed that bran increases PBS-water affinity through reducing the degree of crystallinity, as demonstrated in the undertaken DSC tests. This effect comes about because moisture is more effectively diffused in the polymer amorphous phase than in the crystalline phase. Despite polymer matrix hydrophilicity, the presence of water may have an adverse effect, and, in the case of PBS, induce the hydrolysis of the ester bonds forming the macromolecules. This phenomenon may lead to the deterioration of mechanical properties and thermal resistance, while shortening the degradation time [52,57,60,90].

## 4. Conclusions

The conducted studies confirmed the significant impact of extruder screw rotational speed and bran content in the blend on its extrusion process and selected properties. Our work demonstrated that increasing the rotational speed of the processing screws in the used twin-screw co-rotating extruder results in enhanced screw drive torque, as well as increased mass flow rate and pressure of the extruded blend. A similar effect of processing screw rotational speed on the aforementioned values was demonstrated by other studies involving the extrusion of blends containing plant fillers. In contrast, increasing the screw speed leads to reduced specific energy consumption, which results from the autothermal effect—the blend heating up due to internal friction—and the increase in its flow rate being greater than the reduction in the flux of energy supplied to the extruder. Moreover, increasing the wheat bran content in a blend leads to decreasing its mass flow rate and the screw drive torque, while the pressure of the extruded blend increases. This effect is associated with both increased melt viscosity of the blend as well as with the increasing quantity of water vapor released during bran processing.

The outcome of our microscopic observations indicated the presence of numerous pores in pellets containing wheat bran. Here, their size and quantity depended on the filler content. Their formation is due to structurally-bound water in the filler, the presence of which was indicated in the FTIR and TG tests. As all obtained pelletized biocomposites are only semi-finished products that are subject to further processing, the presence of pores is not a significant drawback.

The FTIR analysis enabled the detection of chemical structures typical for wheat bran building substances and wheat grain endosperm fragments. No presence of chemical bonds at the PBS/bran interface was found.

DSC test results showed the significant impact of bran presence on the degree of crystallinity and the PBS crystallization temperature. We saw that the degree of crystallinity of PBS/WB biocomposites decreased relative to that of pure PBS. This can affect the mechanical and thermal properties of a biocomposite and increase exposure to the influence of water through increasing the share of the amorphous phase, which is more susceptible to water diffusion. In contrast, the crystallization temperature in the course of the cooling of the biocomposites was higher than that for PBS alone. It can, therefore, be concluded that bran acts as a crystallization promoter, while simultaneously limiting crystallite growth.

Increasing bran content results in a clear deterioration of the thermal resistance due to a significantly lower PBS/WB biocomposite decomposition temperature (when compared to neat PBS). A relationship between increased rotational speed and decreased biocomposite thermal resistance was also observed. Improved homogenization, as well as the thermomechanical and thermo-oxidative degradation of PBS macromolecules, may be the reasons for the aforementioned.

Studies involving the melt flow rate index indicated the adverse impact of bran content on biocomposite processability. This results from increased melt viscosity due to the presence of the dispersed phase. At the same time, we found that the processing screw rotational speed in the course of plasticizing and associated shearing (which leads to transforming screw rotational motion mechanical energy into thermal energy) impacted upon the processability of the PBS/bran blend.

Despite the high porosity of the obtained pellets, density tests enabled the identification of the significant positive impact of both bran content and screw rotational speed on the pellet density.

In addition to the above, the results of tests involving the relationship between pressure *p*, specific volume *v* and temperature *T* confirmed the impact of bran content on increased PBS crystallization temperature. These also uncovered the positive impact of the filler on dimensional stability in the course of cooling and depressurization, which in practice translates to lower processing shrinkage.

Finally, we noted that the strong affinity of wheat bran to water caused increased air-moisture absorbance along with increasing bran content. This necessitates drying the pellet directly prior to further processing, since the studies demonstrated the rapid growth of moisture content after 24 h post-drying.

## Figures and Tables

**Table 1:** Experimental design and experimental test results – mean values.

Experimental Design Layout	*n*, min^−1^	*u*, %	*G*, g/s	*ρ*, g/cm^3^	*MFR*, g/10 min	*M*, Nm	*P*, MPa	*E_jc_*, J/g
1	72	15.9	3.32	1.1137	1.36	76.8	4.63	1443
2	72	44.1	2.68	1.2101	0.40	50.8	6.76	1409
3	178	15.9	6.99	1.1668	1.38	80.9	6.30	1002
4	178	44.1	5.87	1.2693	0.58	69.6	8.96	1053
5	50	30.0	2.19	1.2130	0.92	58.3	4.75	1706
6	200	30.0	7.11	1.2307	0.93	71.9	7.83	935
7	125	10.0	5.20	1.1117	1.54	82.0	5.12	1179
8	125	50.0	3.58	1.3307	0.27	58.7	8.93	1135
9–13 (C)	125	30.0	4.76	1.2145	0.72	69.2	6.78	1117

**Table 2:** Model of polymer mass flow rate *G*—ANOVA table, R^2^ = 0.99; R_adj_^2^ = 0.99.

Source of Variation	SS	df	MS	F	*p*
*n*	119.4340	1	119.4340	4579.34	0.0000
*u*	8.3836	1	8.3836	321.44	0.0000
*u* ^2^	0.3020	1	0.3020	11.58	0.0015
n*u	0.2856	1	0.2856	10.95	0.0019
Error	1.0954	42	0.0261		
Total SS	129.2426	46			

SS—sum of squares, df—number of the degrees of freedom, MS—mean sum of squares, F—values of the test statistic, *p*-value of probability corresponding to the test statistic value.

**Table 3:** Model of screw drive torque M—ANOVA table, R^2^ = 0.97; R_adj_^2^ = 0.96.

Source of Variation	SS	df	MS	F	*p*
*n*	222.7669	1	222.7669	65.54	0.00004
*n* ^2^	21.7453	1	21.7453	6.40	0.03529
*u*	615.7020	1	615.7020	181.14	0.00000
n*u	53.5098	1	53.5098	15.74	0.00413
Error	27.1928	8	3.3991	-	-
Total SS	940.9027	12	-	-	-

SS—sum of squares, df—number of the degrees of freedom, MS—mean sum of squares, F—values of the test statistic, *p*-value of probability corresponding to the test statistic value.

**Table 4:** Model of polymer blend pressure *P*—ANOVA table, R^2^ = 0.99; R_adj_^2^ = 0.99.

Source of Variation	SS	df	MS	F	*p*
*n*	8.475891	1	8.475891	749.89	0.000000
*n* ^2^	0.421195	1	0.421195	37.26	0.000488
*u*	12.980035	1	12.980034	1148.39	0.000000
*u^2^*	0.105562	1	0.105562	9.34	0.018425
*nu*	0.068909	1	0.068909	6.10	0.042887
Error	0.079120	7	0.011303	-	-
Total SS	22.195700	12	-	-	-

SS—sum of squares, df—number of the degrees of freedom, MS—mean sum of squares, F—values of the test statistic, *p*-value of probability corresponding to the test statistic value.

**Table 5:** Model of specific energy consumption of the extruder *E_jc_*—ANOVA table. R^2^ = 0.93; R_adj_^2^ = 0.92.

Source of Variation	SS	df	MS	F	P
*n*	445,039.6	1	445,039.6	116.1	0.000000
*n* ^2^	65,932.3	1	65,932.3	17.2	0.001988
Error	38,329.7	10	3833.0	-	-
Total SS	549,290.5	12	-	-	-

SS—sum of squares, df—number of the degrees of freedom, MS—mean sum of squares, F—values of the test statistic, *p*-value of probability corresponding to the test statistic value.

**Table 6:** Melting point *T_m_*, crystallization *T_c_* and glass transition *T_g_* temperatures, the enthalpy of melting Δ*H_m_* and degree of crystallinity *X_c_* of PBS and its composites with bran, based on differential scanning calorimetry (DSC) thermograms.

Sample	Heating I				Cooling	Heating II			
	*T_g_* [°C]	*T_m_* [°C]	Δ*H_m_* [J/g]	*X_c_* [%]	*T_c_* [°C]	*T_g_* [°C]	*T_m_* [°C]	Δ*H_m_* [J/g]	*X_c_* [%]
PBS	−30.0	123.4	95.1	86.2	78.4	−30.7	119.2	78.7	71.4
1(16)	−31.2	121.5	68.5	73.9	86.4	−30.2	120.3	47.1	50.8
2(44)	−29.4	121.2	42.8	69.3	81.8	−28.1	121.1	28.9	46.8
3(16)	−29.7	124.8	71.3	77.0	84.9	−29.7	120.8	55.0	59.4
4(44)	−28.9	122.4	53.8	87.1	81.9	−28.5	121.1	36.4	58.9
5(30)	−31.3	119.9	57.3	74.2	84.5	−29.4	119.9	36.1	46.8
6(30)	−31.3	120.9	62.5	80.9	82.5	−30.7	120.1	41.3	53.5
7(10)	−31.0	121.5	76.2	76.8	87.5	−30.4	119.9	55.0	55.4
8(50)	−30.5	119.1	41.7	75.6	83.8	−29.3	119.7	26.7	48.4
9(30)	−31.4	120.7	55.8	72.3	83.7	−31.8	120.6	40.5	52.5

**Table 7:** Parameters characterizing the thermal resistance of PBS, bran and biocomposites, obtained based on thermogravimetry (TG) and derivative thermogravimetry (DTG) curves.

	*T*_5%_ [°C]	*T*_50%_ [°C]	*T_max_*_1_ [°C]	Δ *m*_1_ [%]	*T_max_*_2_ [°C]	Δ *m*_2_ [%]	*T_max_*_3_ [°C]	Δ *m*_3_ [%]	*R_m_* [%]
bran	201	303	296	68.0	-	-	459	29.7	2.3
PBS	329	390	-	-	399	94.3	502	2.8	2.9
1(16)	294	385	305	12.4	392	81.0	480	6.4	0.2
2(44)	261	376	300	27.4	389	59.6	450	12.2	0.8
3(16)	292	386	304	11.5	393	82.0	480	6.4	0.1
4(44)	260	372	300	30.9	387	55.9	462	12.7	0.5
5(30)	286	388	303	17.6	401	71.1	484	8.4	2.9
6(30)	271	380	303	20.9	394	70.7	472	8.2	0.2
7(10)	303	389	308	7.5	396	87.6	492	4.8	0.1
8(50)	260	372	299	29.9	387	57.2	460	12.7	0.2
9(30)	275	381	303	19.6	390	70.6	479	9.6	0.2

**Table 8:** Model of melt flow rate (*MFR*)—ANOVA table. R^2^ = 0.99; R_adj_^2^ = 0.99.

Source of Variation	SS	df	MS	F	*p*
*n*	0.027464	1	0.027464	29.25	0.000003
*n* ^2^	0.159403	1	0.159403	169.77	0.000000
*u*	7.952753	1	7.952753	8470.05	0.000000
*u* ^2^	0.125630	1	0.125631	133.80	0.000000
*nu*	0.033620	1	0.033620	35.81	0.000001
Error	0.036618	39	0.000939	-	-
Total SS	8.226770	44	-	-	-

SS—sum of squares, df—number of the degrees of freedom, MS—mean sum of squares, F—values of the test statistic, *p*-value of probability corresponding to the test statistic value.

**Table 9:** Model of density *ρ*—ANOVA table. R^2^ = 0.88; R_adj_^2^ = 0.87.

Source of Variation	SS	df	MS	F	P
*n*	0.007075	1	0.007075	11.81	0.00215
*u*	0.096988	1	0.096988	161.93	0.00000
Error	0.014374	24	0.000599	-	-
Total SS	0.118437	26	-	-	-

SS—sum of squares, df—number of the degrees of freedom, MS—mean sum of squares, F—values of the test statistic, *p*-value of probability corresponding to the test statistic value.

## Data Availability

The data presented in this study are available on request from the corresponding author.

## Abbreviations and Symbols

PBS	Poly(butylene succinate)
WB	Wheat bran
DOE	Design of experiment
*MFR*	Melt flow rate
FTIR	Fourier transform infrared (spectroscopy)
DSC	Differential scanning calorimetry
*X_c_*	Degree of crystallinity
Δ*H_m_*	Melting enthalpy
*T_m_*	Melting point
*T_c_*	Crystallization temperature
*T_g_*	Glass transition temperature
TG	Thermogravimetry
DTG	Derivative thermogravimetry
*T_5%_*, *T_50%_*	Temperature of 5% and 50% of mass loss
*T_max_*	Temperature of the maximum rate of mass loss
Δ *m*	Mass loss corresponding to *T_max_*
*R_m_*	Residual mass
*m*	Mass of the extrudate
*M*	Screw drive torque
*P*	Polymer pressure in the plasticizing system
*Q*	Total electric power supplied to the extruder
*G*	Mass flow rate
*E_jc_*	Specific energy consumption
*p-v-T*	Relationship between pressure *p*, specific volume *v* and temperature *T*
*ρ*	Density
*u*	Wheat bran content
*n*	Screw rotational speed
